# The future of surgery: A case of Schatzker type II tibial plateau fracture operated with arthroscopic-assisted technique

**DOI:** 10.1016/j.ijscr.2024.110107

**Published:** 2024-07-31

**Authors:** Jeffry Andrianus, Muhammad Rifqi Farizan Akbar, Farrel Aqila Yahya

**Affiliations:** a Department of Orthopaedics and Traumatology, Faculty of Medicine, Universitas Airlangga, Surabaya, Indonesia; b Department of Orthopaedics and Traumatology, Dr. Soetomo General Academic Hospital, Surabaya, Indonesia; c Faculty of Medicine, Universitas Airlangga, Surabaya, Indonesia

**Keywords:** Tibial plateau fractures, Schatzker type II tibial plateau fractures, Arthroscopic-assited surgery

## Abstract

**Introduction and importance:**

One percent of adult fractures are tibial plateau fractures, but can represent significant morbidity for patients. Achieving anatomic reduction of the articular surface, adequate alignment, stable fixation consistent with early mobilization, and minimal soft tissue injury are the key goals of treatment. Compared to open reduction and internal fixation, the decreased invasiveness of arthroscopy-assisted percutaneous fixation translates into decreased morbidity rates.

**Case presentation:**

A 35-year-old woman lost control of motorcycle and landed on her left knee. Immediate pain in her left knee and was unable to ambulate or move her knee. Initial radiographs showed a depressed lateral tibial plateau fracture and from computed tomography (CT) scan showed a depressed posterolateral tibial plateau fracture with incongruence of his joint space. Classifying the injury as a Schatzker type 2 tibial plateau fracture She underwent an arthroscopic-assisted open reduction internal fixation of her lateral tibial plateau.

**Clinical discussion:**

Various surgical methods are available for treating tibial plateau fractures, including open, fluoroscopic-assisted, and arthroscopic approaches. Promptly addressing depressed articular surfaces is crucial to prevent rapid arthrosis progression. Arthroscopic-assisted procedures offer benefits like direct visualization of reduction, treatment of intra-articular issues, and faster patient recovery. Recent advancements in arthroscopic techniques enable precise reduction without fluoroscopy, reducing soft tissue damage and the risk of complications such as infection and cartilage damage.

**Conclusion:**

Arthroscopic-assisted surgery offers precise treatment for Schatzker type II tibial plateau fractures, representing a promising future direction in surgery.

## Highlights

• Arthroscopic-assisted procedures offer benefits like direct visualization of reduction.
• Arthroscopic techniques enable precise reduction without fluoroscopy.
• Arthroscopic techniques enable to get optimal anatomical reduction.

## 1 Introduction

Fractures of the tibial plateau is a significant challenge for many orthopedic surgeons although comprise around 1 % of all fractures, but can represent significant morbidity for patients [1,2]. The prevalence of tibial plateau fractures is estimated to be around 10.3 per 100,000 person-years. The prevalence of this occurrence is greater in males than in females, and the likelihood of it happening rises with age, especially in those over the age of 50 as a result of osteoporotic alterations [3]. Achieving anatomic reduction of the articular surface, adequate alignment, stable fixation consistent with early mobilization, and minimal soft tissue injury are the key goals of treatment for this frequently difficult surgical case [3]. Compared to open reduction and internal fixation, the decreased invasiveness of arthroscopy-assisted percutaneous fixation translates into decreased morbidity rates [4]. Studies have indicated that when it comes to Schatzker types I, II, and III fractures, arthroscopy assisted procedures can prove to be more efficacious than more intricate fracture patterns. From the clinical and supporting features, a preoperative overview and plan is obtained to assess and plan the actions to be taken. The incidence of union is often high for Schatzker Type II fractures treated arthroscopically, with reported rates typically ranging from 90 to 95 % [4]. The incidence of complications, such as infections, non-unions, or the requirement for additional procedures, is often minimal, with rates ranging from 5 to 10 %. A significant number of patients are able to resume their pre-injury levels of activity within 6–12 months after undergoing surgery, with a high rate of returning to work or sports, typically ranging from 80 % to 90 % [5,6].

Compared to unicondylar fractures, bicondylar fractures typically result in worse outcomes. Bicondylar fractures tend to show worse results than unicondylar fractures [5]. During arthroscopic procedures, if the cortical envelop cannot be easily restored, then open techniques are prudent. Comminuted fractures and Schatzker types IV to VI are generally not recommended for treatment with arthroscopic techniques because of increased risk of fluid extravasation, which could lead to a compartment syndrome [5]. For depressed lateral tibial plateau fractures, precise chondral reduction can only be achieved with arthroscopic-assisted operative treatment. Anatomic reduction is therefore of utmost importance [6]. However, achieving chondral surface reduction necessitates direct visualization during the reduction process. Our case highlights the efficacy of this approach and its potential for future advancement. We constructed this Case Report Using SCARE Guideline [7].

## 2 Case presentation

**Fig. 1:**
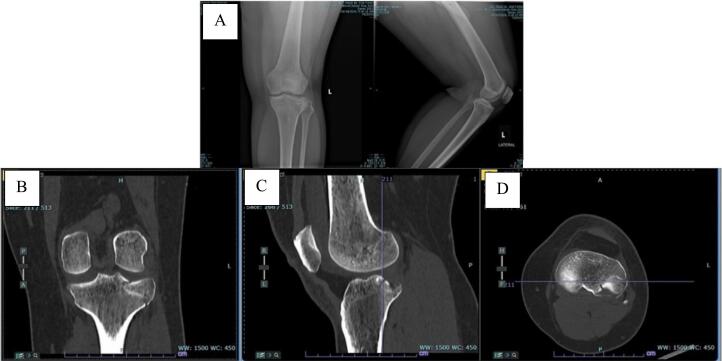
This Picture shows Xray and CT-Scan of left knee before performing surgery. The picture shows knee from some position. A) Xray AP/Lat of the Left Knee, B) Coronal CT Scan, C) Sagittal CT Scan, and D) Axial CT axial.

A 35-year-old woman lost control of her motorcycle after avoiding a car that stopped suddenly and landed on her left knee. She had immediate pain in her left knee and was unable to ambulate or move her knee. In the scene, she was noted to have a large effusion of her left knee. Her initial radiographs showed a depressed lateral tibial plateau fracture and from computed tomography (CT) scan (Fig. 1) showed a depressed posterolateral tibial plateau fracture with incongruence of his joint space. Once an agreement is reached, the patient will personally sign in order to acknowledge any potential outcomes resulting from their condition or treatment, including the side effect of doing surgery for her case.

**Fig. 2:**
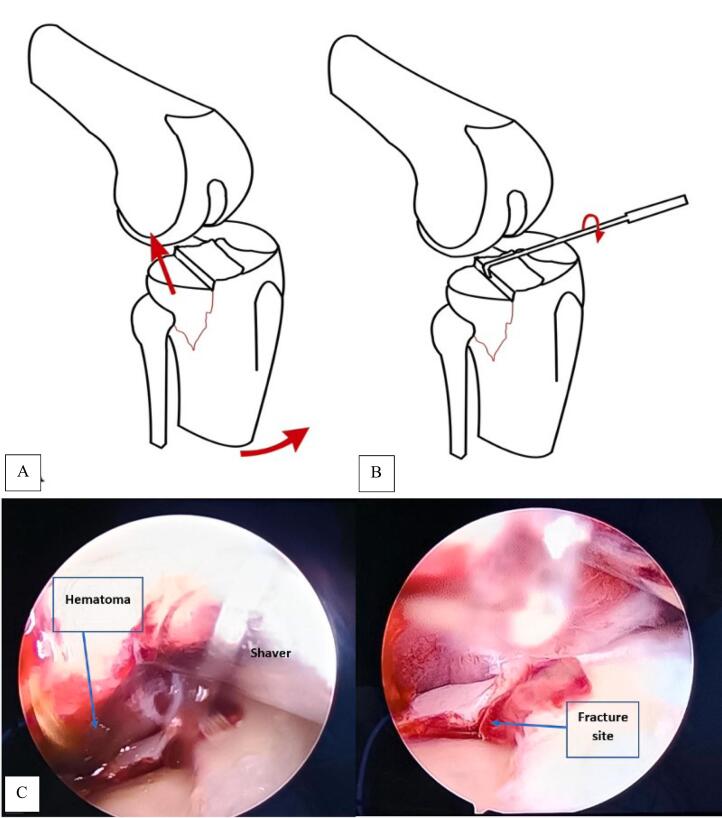
First identification spot of fracture site. A) Fracture reduction via ligament traction: the traction exerted on the bone structures elevates the fragment. B) Use of a palpation hook to dis-impact the fracture [12]. C) Arthroscopic view to see the fracture site.

She underwent an arthroscopic-assisted open reduction internal fixation of her lateral tibial plateau. After obtaining an anterolateral portal a 3-compartment diagnostic examination was completed, which demonstrated the depressed fracture of the posterolateral tibial plateau. An anteromedial portal was made to introduce tools and her medial meniscus was closely examined with a probe and no tear was identified (Fig. 2).

The operative procedure for Arthroscopic including; preoperative preparation ➔ patient positioning ➔ diagnostic arthroscopy ➔ arthroscopic-assisted fracture reduction ➔drill tunnel

to depressed site fracture ➔ elevating at the depressed fragment with bone graft to make congruence joint surface ➔ fixation ➔ assessment and final adjustments ➔ closure ➔ postoperative care [3].

The long and laborious process of hematoma evacuation precedes the actual surgery (Fig. 2). A cannula that aids in hematoma evacuation and lowers the possibility of excessive joint pressure can be introduced through a third port placed superolateral. A shaver can be inserted into the joint cavity to help remove clots and tiny bone fragments once intra-articular visibility is adequate. The instruments in the ports can be moved around to suit your needs [8].

From the clinical and supporting features, a preoperative overview and plan is obtained to assess and plan the actions to be taken. Before performing the surgery, arthroscopy was used to diagnose the knee. The knee was positioned figure of four to reach the posterolateral position. The fracture site was identified and the posterolateral tibial plateau depressed [9].

**Fig. 3:**
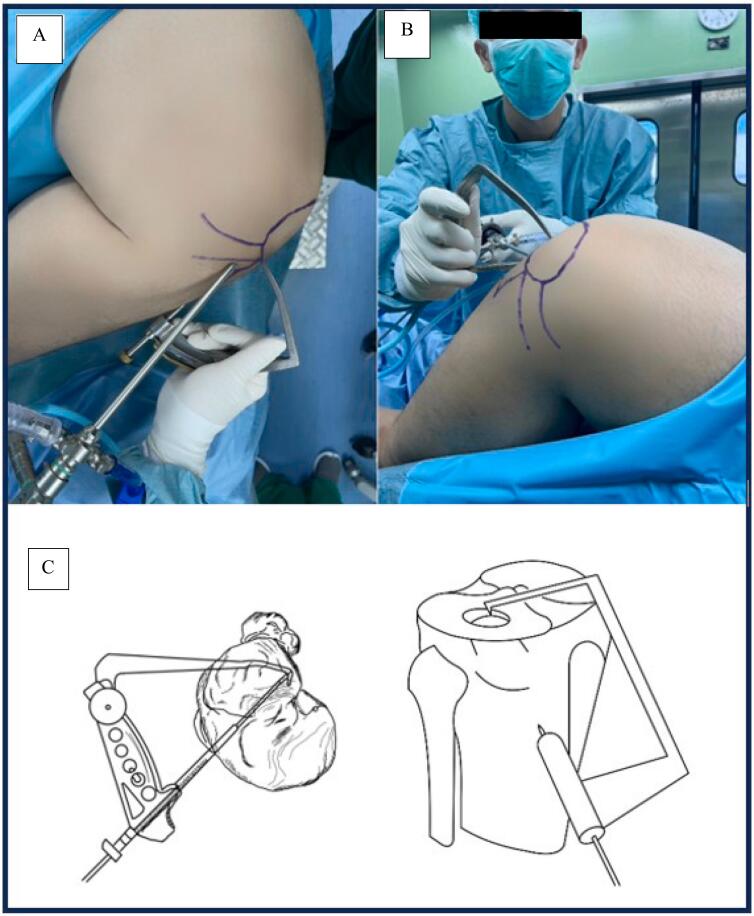
Procedure which is done to get direction to fracture site. A) The anterior cruciate ligament tibial drill guide is inserted via the anterolateral portal, and the tip of the drill guide is placed at the depressed articular fragment. B) the 6-mm cannulated reamer is used to open the anteromedial cortex of the tibia. C) Illustration to get direction to fracture site [4].

The next step was to identify the normal lateral margin using arthroscopy (Fig. 3A). ACL Tibia drill guide tunnel of ACL was inserted through the anterolateral portal to marker the depressed site. A step incision is then made on the anterior tibia to insert the cannula or trocar. The wire is placed into the marker ACL Tibia drill guide tunnel, then the hook is used as a marker direction to the depressed area. Drilling was then performed with a 5.0 tunnel ACL drill bit and enlarged with an 8.0 drill bit. Drilling was only done until the subchondral did not penetrate the articular (Fig. 3).

Applying the elevating force at the depression's centre is essential for a high-quality decrease of the depressed joint surface. In order to achieve this, a ligamentoplasty aiming system should be used to place a guiding pin in the centre of the depression [1]. In order to produce a fragment-elevating force that is almost perpendicular to the joint surface and prevent damage to the normal condyle, the pin can be made to pass through the anterior cortex of the fractured condyle and into the bone. As an alternative, a bigger volume of cancellous bone can be obtained by inserting the pin through the other condyle in order to elevate the fragment [8].

Nevertheless, this alternate method damages the intact condyle and raises the joint surface in a more tangential direction; it might take some manoeuvring to correct the reduction. By using a cross-sighting technique, we have not had any cases where the pin has broken through the condyle. A ligamentoplasty auger directed across the cancellous bone to produce the anterior cortical window for access [9]. It can utilise the pin that was previously placed into the depression. A trephine with a diameter of 10 mm is inserted manually or with the use of a power tool to a depth of 2 cm beneath the depression. This removes the cancellous bone core, which is subsequently gently packed in subchondral using a bone tamp with a diameter of 8 mm [10,11].

**Fig. 4:**
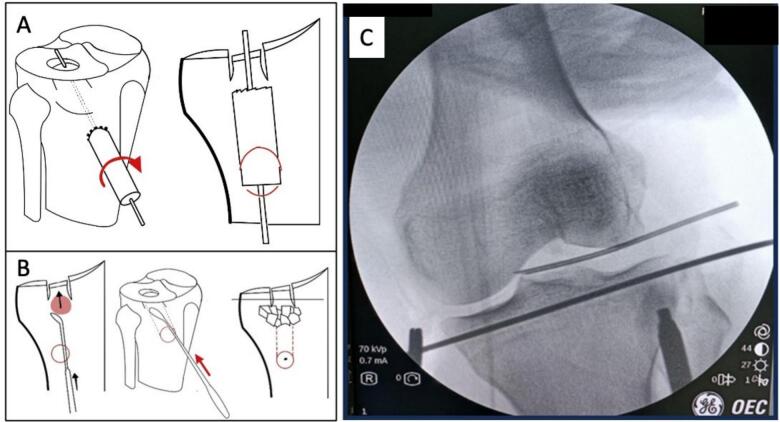
A) This is the illustration to make a tunnel, under the depression area [4]. B) Illustration to fill tunnel with bone graft [3]. C) X-ray picture when bone graft is filled.

The guiding pin can be used to raise the fragment if an impactor or cannulated bone tamp is available. If not, the maneuver becomes less reliable since the guide-pin needs to be taken out. Under the supervision of an arthroscopic and fluoroscopic physician, elevation should be done extremely carefully. Using a spatula, adjustments can be made to the decrease of the depressed fragment's rim as desired. To enable the femoral condyle to shape the joint surface, it is preferable to slightly overcorrect the joint surface depression, which is followed by knee flexion (Fig. 4) [12].

Through the insertion of one or two pins one centimetre below the joint surface, temporary stabilization is accomplished. Anteroposterior and lateral fluoroscopy images should be used to assess pin location. An automatic pinning system was uses a targeting frame to guide the fixation wires just under the previously elevated joint surface by sliding over the pin that is implanted into the middle of the elevated fragment. In mixed fractures (Schatzker type 2) that exhibit both depression and cleavage [8]. It is best to combine the technical strategies that have already been discussed. Using a large bone-grasping forceps, the depressed fragment is lifted first, and the separation is subsequently minimised.

Final step was all instruments were removed and the bone graft was inserted from the tunnel to evaluate the depressed area using a draper until it rose. The articular surface was then evaluated using arthroscopy to assess whether the depressed area was re-aligned. The ACL Tibia drill guide was placed horizontally, and an incision was made at the medial tibial plateau. An anterolateral proximal tibia incision was then made. The ACL Tibia drill guide was then placed medially using a drill. After that, the wire was attached and drilling (cannulated drill) size 7.0 was performed. A single cannulated screw size 7.0 was then inserted. Finally, an evaluation was performed using the C arm (Fig. 4).

**Fig. 5:**
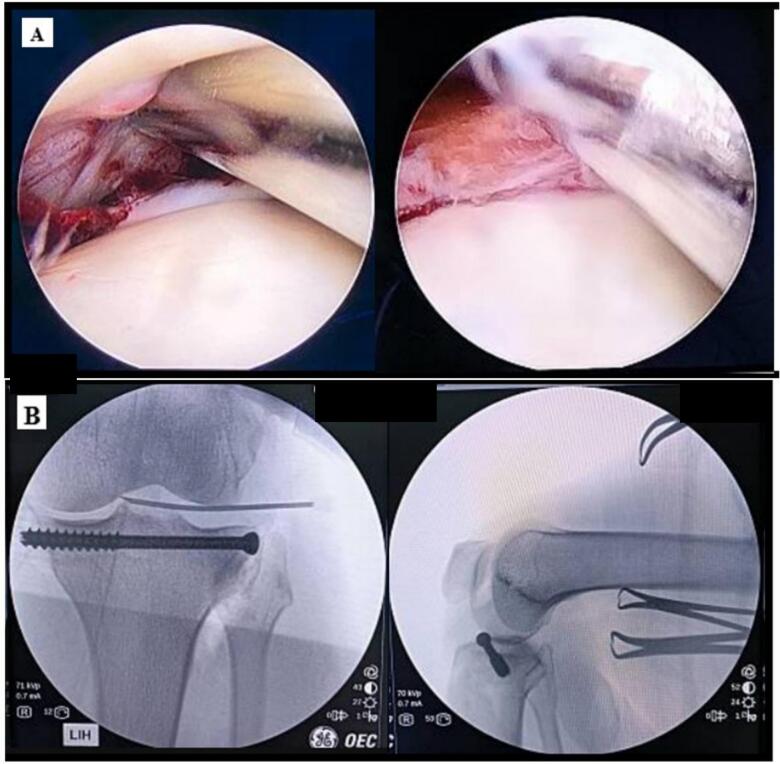
Final Post operative evaluation after surgery. A) Picture from arthrocopy. B) Picture from xray.

Final post operative evaluation after surgery was applied. Static quadriceps exercises with passive knee range of motion are then recommended. For at least eight weeks, the patient is non-weight bearing and uses axillary crutches to walk. During the first two weeks, wound healing is evaluated. The patient has three to four months to resume full exercise (Fig. 5).

Periodic postoperative evaluation was performed by clinical and radiological follow-ups to monitor the patient's healing process and functional outcomes. Postoperative care and rehabilitation are essential aspects of the recovery process after arthroscopic-assisted surgery for tibial plateau fractures. By following procedures, patients can attain the best possible results, which include the restoration of function, the reduction of discomfort, and the enhancement of their quality of life. A comprehensive and successful rehabilitation requires the involvement of surgeons, physical therapists, and other healthcare experts in a multidisciplinary approach.

## 3 Discussion

Tibial plateau fractures have been reported to be treated surgically using a variety of methods, including open, fluoroscopic-assisted, and arthroscopic methods. If treatment is not applied to the depressed articular surface, arthrosis will advance quickly [1]. Using an ACL tibial drill guide and anterior-to-posterior screws for internal fixation, Ackermann et al. suggested an arthroscopic controlled reduction of the posterolateral tibial plateau depressed fracture [8]. Alvarez et al. explain step with a lateral incision and reduction by tamping into the fracture site described successful arthroscopic reduction of a split-depressed tibial plateau fracture [9]. Acute ACL injury and a depressed posterolateral tibial plateau fracture were later treated arthroscopically, as reported by Park et al. [10]. Then, to stabilize the depressed fracture, the decreased fracture is repaired in situ with a press-fit fibular allograft. The technique being discussed has several benefits, such as a reduction in soft tissue dissection, direct visualization of fracture reduction, absence of donor site morbidity, no requirement for hardware implant fixation, no need for fluoroscopy, and longer operating times. With reduced iatrogenic articular cartilage damage and the use of the ACL tibial drill guide, this approach improves the precision of fracture reduction. An anatomic decrease of articular cartilage and an associated intraarticular lesion are confirmed by direct visualization using an arthroscope. The minimally invasive method reduces the risk of infections and wound complications. The current method fulfils bone defects without requiring implants for fracture stabilization, nor does it cause donor-site morbidity from iliac crest graft harvest. [11]

This procedure is performed with typical knee arthroscopy tools while the patient is supine. In summary, the surgical approach that has been demonstrated here is safe and reproducible for the treatment of depressed lateral tibial plateau fractures. There are many advantages to surgery using arthroscopic assisted internal fixation compared to the disadvantages of tibial plateau fracture surgery [14] (Table 1). After surgery, patients can usually resume bearing weight within 6 to 12 weeks. However, it may take several months for them to fully heal and resume their normal activities. In certain cases, patients may need up to 6 months to restore full strength and function in the damaged limb. After surgery, patients frequently return to near-normal or adequate range of motion in the first few weeks or months. As recovery advances, more improvement in range of motion should be anticipated. Compared to open operations, arthroscopic procedures usually have lower infection rates typically less than 5 % [13]. Prophylactic antibiotics and strict sterile procedures help reduce this danger.

**Table 1:** Advantages and disadvantages of Arthroscopic assisted internal fixation in tibial plateau fracture [11].

Advantages	Disadvantages
Less soft-tissue dissection and time of recovery	Need arthroscopic experience
Direct visualization of fracture reduction through an arthroscope	Availability of fresh-frozen femoral head allograft
No donor-site morbidity	Risk of disease transmission/infection
No need for hardware implant fixation	Need further immobilization
No need for fluoroscopy	Risk of compartment syndrome from fluid extravasation
Less operative time	

In the current management of tibial plateau fractures, arthroscopy plays a crucial role. Thorough knowledge of arthroscopy is essential. With arthroscopy, fracture reduction can be assessed without requiring a large incision for an arthrotomy. It allows for the best possible care of concurrent lesions. The best results from arthroscopy are obtained with stable percutaneous fixation that allows for quick mobility [14]. Thus, fractures of the lateral tibial plateau that are pure split, pure depression, or split-depression provide the greatest signals. A skilled surgeon can reduce surgical trauma in patients with complicated proximal tibial fractures by using arthroscopy [12]. The short- and medium-term results were favorable in the majority of investigations. Studies with follow-ups longer than three years were rare, though. After more than five years of follow-up, Cassard et al. reported knee and function scores above 90 %, while Scheerlinck et al. found that 79 % of patients had outstanding HSS knee ratings. In 63 % of the patients evaluated by Scheerlinck et al. and 87 % of patients in a research by Holzach et al., a return to the prior level of sporting activities was achieved [14]. It should be noted that 10 % to 30 % of patients followed up for more than three years had significant joint space normalcy. While recovery times, ranges of mobility, and complication rates from arthroscopic treatments for tibial plateau fractures appear encouraging, research should carefully examine constraints such sample size, selection bias, and generalizability of findings [14]. Furthermore, determining how cost-effective these approaches are in comparison to more conventional ones is essential for guiding resource allocation and healthcare decision-making. These factors should be taken into account in future research by using carefully planned prospective studies with sufficient sample sizes and long-term follow-up [14].

## 4 Conclusion

The results we achieve indicate that implementing the arthroscopic-assisted approach led to favorable outcomes and enhanced functionality. The implementation of arthroscopic procedures for tibial plateau fracture surgery has the capacity to greatly influence both healthcare systems and the quality of life for patients. Arthroscopy can make the healthcare system more sustainable and offer significant advantages to patients by lowering costs, increasing efficiency, and improving patient outcomes. This can lead to faster recoveries and higher levels of patient satisfaction. Periodic postoperative assessments verified the successful wound healing and the exceptional restoration of the patient's functionality.

## Consent

Written informed consent was obtained from the patient for publication of this case report and accompanying images. A copy of the written consent is available for review by the Editor-in-Chief of this journal on request.

## Ethical approval

Regarding to the observational study of outcome in our case report, the ethical approval was waived by the Ethical Committee of Dr. Soetomo General Academic Hospital, Surabaya, Indonesia. However, the copies of informed consent are available for review by the Editor-in-Chief of this journal on request.

## Funding

This case report received no specific grant from any funding agency in the public, or non-profit sector.

## Author contribution

Jeffry Andrianus involved in performing surgical technique, conceptualization, data curation, investigation, formal analysis, methodology, visualization, project administration, writing-review and editing.

Muhammad Rifqi Farizan Akbar involved in performing surgical technique, data curation, investigation, formal analysis, methodology, visualization, project administration, writing-original draft.

Farrel Aqila Yahya involved in formal analysis, project administration, data curation, writing-review and editing

## Guarantor

Jeffry Andrianus.

## Research registration number

N/A.

## Conflict of interest statement

Jeffry Andrianus: Concepts, designer, definition of intellectual content, literature search, clinical studies, data acquisition, data analysis, manuscript preparation, manuscript preparation, manuscript editing, manuscript review, Guarantor

Muhammad Rifqi Farizan Akbar: Concepts, designer, definition of intellectual content, literature search, clinical studies, manuscript preparation, manuscript review

Farrel Aqila Yahya: Concepts, design, literature search, data acquisition, data analysis, manuscript editing.

## References

[1] Krause M., Preiss A., Meenen N.M., Madert J., Frosch K.H. (Aug 2016). “Fracturoscopy” is superior to fluoroscopy in the articular reconstruction of complex tibial plateau fractures—an arthroscopy assisted fracture reduction technique. J. Orthop. Trauma.

[2] Chilmi M.Z., Febrian F. (Apr 30 2024). The application of posterior plate in Tibial Plateau fracture: a case report. J. Orthopaed. Traumatol. Surabaya.

[3] Zuo Chen X., Gang Liu C., Chen Y., Qiang Wang L., Zheng Zhu Q., Lin P. (Jan 2015). Arthroscopy-assisted surgery for Tibial plateau fractures. Arthroscopy.

[4] Burdin G. (Feb 2013). Arthroscopic management of tibial plateau fractures: surgical technique. Orthop. Traumatol. Surg. Res..

[5] Ohdera T., Tokunaga M., Hiroshima S., Yoshimoto E., Tokunaga J., Kobayashi A. (Nov 1 2003). Arthroscopic management of tibial plateau fractures?Comparison with open reduction method. Arch. Orthop. Trauma Surg..

[6] Hermanowicz K., Malinowski K., Góralczyk A., Guszczyn T., LaPrade R.F., Sadlik B. (Jun 2019). All-arthroscopic treatment of Schatzker type III lateral Tibial plateau fracture without fluoroscopy. Arthrosc. Tech..

[7] Sohrabi C., Mathew G., Maria N., Kerwan A., Franchi T., Agha R.A. (May 2023). The SCARE 2023 guideline: updating consensus surgical case report (SCARE) guidelines. Int. J. Surg..

[8] Ackermann C., Frings J., Alm L., Frosch K.H. (Aug 2019). Arthroscopic controlled closed reduction and percutaneous fixation of posterolateral tibia plateau impression fractures. Arthrosc. Tech..

[9] Alvarez A., Youn G.M., Remigio Van Gogh A.M., Shin Yin S.S., Chakrabarti M.O., McGahan P.J. (Feb 2020). Tibial Plateau With Arthroscopic Reduction–Internal Fixation. Arthrosc. Tech..

[10] Park J.P., Laverdière C., Corban J., Böttcher J., Burman M.L., Martin R. (Sep 2020). An arthroscopic procedure for restoration of posterolateral Tibial plateau slope in Tibial plateau fracture associated with anterior cruciate ligament injuries. Arthrosc. Tech..

[11] Itthipanichpong T., Kuptniratsaikul S., Limskul D., Thamrongskulsiri N. (Jun 2022). Arthroscopic-assisted reduction of depressed lateral Tibial plateau fracture using precision Drill guide and fresh-frozen femoral head allograft. Arthrosc. Tech..

[12] Cassard X., Beaufils P., Blin J.L., Hardy P. (1999). Osteosynthesis under arthroscopic control of separated tibial plateau fractures. Rev. Chir. Orthop..

[13] Scheerlinck T., Ng C.S., Handelberg F., Casteleyn P.P. (1998). Medium-term results of percutaneous, arthroscopically assisted osteosynthe- sis of fractures of the tibial plateau. J. Bone Joint Surg. Br..

[14] Łoś D., Nowak A., Dziedziński D., Janus A., Kaptur A. (2024). Use of arthroscopy in management of tibial plateau fractures. Qual. Sport.

